# Insight into the Spectrum of Coronary Atherosclerosis in Asymptomatic Urban Han Chinese Population by Coronary Computed Tomography Angiography

**DOI:** 10.1371/journal.pone.0132188

**Published:** 2015-07-07

**Authors:** Jiangbing Li, Ruihong Liu, Xiaokang Ji, Hao Xue, Guang Zhang, Chunxia Wang, Qicai Chen, Fuzhong Xue, Lianqun Cui

**Affiliations:** 1 Department of Cardiology, Shandong Provincial Hospital, Shandong University, Jinan, Shandong, China; 2 Division of Biostatistics, School of Public Health, Shandong University, Shandong, China; 3 Department of Neurosurgery, Qilu Hospital, Shandong University, Shandong, China; 4 Health Management Center of Shandong Provincial Qianfoshan Hospital, Jinan, Shandong, China; 5 Health Management Center of Affiliated Hospital of Jining Medical University, Jining, Shandong, China; 6 Health Management Center of Shengli Qilfield Central Hospital, Shandong, China; Morehouse School of Medicine, UNITED STATES

## Abstract

**Objectives:**

Highlighted the spectrum of coronary atherosclerosis in asymptomatic population by Computed Tomography Angiography (CTA) and developed a surrogation of expensive CTA to early detect coronary atherosclerosis.

**Methods:**

Three hundred and seven self-referred urban Han Chinese asymptomatic individuals underwent coronary CTA were consecutively enrolled. Total plaque score (TPS), Segment stenosis score (SSS) and Coronary Artery Disease severity (CADS) were used to measure and illustrate the spectrum of atherosclerosis burden by mapping their incidence and proportion onto coronary artery tree. Logistic regression model was further used to explore the association between lipid biomarkers and TPS (SSS) for developing a surrogation of CTA to early detect coronary atherosclerosis.

**Results:**

We found that the incidence of TPS, SSS and CADS were up to 71.34%, 68.08%, and 71.34%; and high-risk individuals reached up to 11.07%, 15.31% and 16.29% respectively. All TPS, SSS and CADS were much higher in male than female, and have trend of increasing with age. The most lesion segment emerged on proximal LAD, followed by proximal RCA, mid LAD, proximal LCX, and mid RCA with mixed plaque as dominant. HDL-C was a predictor to both TPS [OR: 0.12 (0.02–0.82)] and SSS [OR: 0.15 (0.03–0.76)], and could identify the serious atherosclerosis subjects of TPS or SSS score >5 (AUC 0.73 and 0.70).

**Conclusions:**

The atherosclerosis plaque burden was about one in ten as high-risk individuals in this specific urban Han Chinese population. As potential surrogation of CTA, HDL-C was recognized as a significant predictor to atherosclerosis burden and revealed a good performance for identifying high-risk individuals.

## Introduction

Approximately half of patients with major adverse coronary event (sudden cardiac death occurs or acute coronary syndromes) do not experience any symptoms or warning signs, emphasizing that it is crucial to early detect the subclinical coronary atherosclerosis for early intervention [1,2]. Theoretically, coronary angiography (CAG) is the golden standard for diagnosis of coronary artery disease (CAD) in asymptomatic healthy population, but it is illegal due to its invasion feature. As a silver non-invasion diagnostic technique, coronary computed tomographic angiography (CTA) [3, 4] has been successfully used to detect the coronary atherosclerosis in asymptomatic individuals [5, 6, 7], suggesting it was suitable to provide a better insight about spectrum of atherosclerosis, especially the obstructive CAD, in healthy population [8].

In practice, it is an essential task to clarify the spectrum of coronary atherosclerosis in a specific asymptomatic healthy population for targeted intervention. Illustrating the distribution and determinants of early CAD in asymptomatic healthy population is the cornerstone of public health, and informs policy decisions and evidence-based practice for preventive healthcare. Furthermore, highlighting the spectrum of atherosclerosis (location, severity and characteristic of plaque) in coronary artery at population level is a useful instruction of clinical intervention for early prevention, conducting treatment and judging prognosis. Various CTA-based clinical coronary artery plaque scores had been created for measuring atherosclerosis plaque burden, including Total plaque score (TPS) [9, 10], Segment-stenosis score (SSS) [9], Calcium scoring [11, 12], and coronary artery disease severity (CADS) [10], 3-vessel plaque score [9], etc. Among them, TPS, SSS and CADS were particularly appropriate for reflect the burden of atherosclerosis plaque in asymptomatic healthy population. TPS measured the plaque quantity by summarizing plaques in each segment of coronary artery, while SSS assessed the burden of atherosclerosis by combining plaque quantity with the degree of stenosis [9]. Follow-up studies demonstrated that both TPS and SSS were significant predictors for major adverse cardiac events (MACE) if their score were more than five points [9, 13]. In addition, CADS was defined by the max degree of stenosis among segments of coronary artery, and clinical study indicated that any coronary lumen lesions >50% would be diagnosed as obstructive CAD which is a predictor of hard cardiac events [10, 14,15].

We, therefore, conducted a pilot study by consecutively enrolling adult urban Han Chinese asymptomatic subjects who underwent CTA in routine healthy check-up for insight into the spectrum of coronary atherosclerosis in this specific population from 2010 to 2014. TPS, SSS and CADS were used to illustrate the distribution of coronary atherosclerosis burden, and further clarify the spectrum of atherosclerosis (location, severity and characteristic of plaque) in coronary artery. Furthermore, as serum lipid biomarkers, usually including total cholesterol (TC), high density lipoprotein cholesterol (HDL-C), low density lipoprotein cholesterol (LDL-C), triglyceride(TG), were established as causal predictors for coronary artery atherosclerosis, their association with scores (TPS, SSS, CADS) were explored for attempting to create a simple and inexpensive tool for predicting the burden of coronary atherosclerosis in asymptomatic healthy population.

## Method

### Study Population

Three hundred and thirty one urban Han Chinese underwent CTA using 64-slice scanner for general routine health evaluation were consecutively enrolled from February 2010 and April 2014 in Health Management Centers of Shandong Provincial Qianfoshan Hospital, Affiliated Hospital of Jining Medical University, Shengli Oilfield Central Hospital in Shandong province, China. Every enrolled subjects finished iodine allergy test, which had hypersensitivity to iodine-based contrast agents were excluded. All participates were asked whether they had chest pain or equivalent symptoms according to a Rose angina questionnaire [16]. We excluded 24 who had chest pain or discomfort before enrolment without a diagnostic workup to rule out CAD (n = 4), a history of percutaneous coronary intervention (n = 13), a history of myocardial infarction/angina (n = 7). As a result, a total of 307 (256 male, 51 female) self-referred asymptomatic subjects were finally enrolled. Anthropometric measurements such as height, weight were measured with light clothing without shoes for calculating BMI by weight/height^2^ (kg/m^2^). Obesity was defined as BMI more than 28 (kg/m^2^) [17]. Blood pressure (BP) was measured on the right arm from sitting position after 5 minute rest, chosen the average of three readings as the BP values. Hypertension was defined by a systolic blood pressure (SBP) ≥ 140 mmHg, a diastolic blood pressure (DBP) ≥ 90 mmHg or self-reported administration of antihypertensive medications. Smoking was defined as current smoking or cessation of smoking within 3 months. Among them, 95 subjects refused to offer their blood, and 212 were simultaneously tested the serum lipid (TC, HDL-C, LDL-C and TG). 2 of them failed in fasting blood-glucose (FBG) testing. Diabetes mellitus was defined as FBG more than 7.0 mmol/L or oral anti-diabetic therapy or insulin.

### Ethics Statement

The ethics committee of Medicine School of Shandong University approved the study protocol and all participates provided written informed consents.

### Date Acquisition

Participates with heart rates >70 beats/min were given 25mg oral metoprolol (metoprolol tartrate, AstraZeneca) before imaging acquisition, the targeting heart rate was <65 beats/min. The CTA were performed with a 64-slice scanner (Discovery CT750 HD, GE Healthcare). The initiation non-enhanced scan was fixed at the apices of lung and termination of scan at the level of the diaphragm. A bolus of 80ml iodixanol (370 mgI/ ml) was injected (5ml/s) and followed by 30ml saline solution. A region of interest was fixed in descending thoracic aorta, and image acquisition was automatically initiated once a threshold (120 Hounsfield units HU) had been reached with bolus tracking. The scanning parameters were: tube voltage 120KV, tube current 400mA, slice thickness 0.625mm, Pitch 0.2. The electrocardiogram of participates were simultaneously recorded to allow for retrospective segmental data reconstruction. If there are motion artifacts additional reconstruction were performed.

### Image Analyses

All subjects underwent coronary CTA with a 64-slice scanner, their scans were transferred to dedicated workstation and analyzed by 2 expert readers. For any disagreement between 2 readers, a consensus interpretation was achieved to obtain a final diagnosis involving a third expert reader. All participates have normal sinus rhythm and capable of holding breath to meet the needing for CTA. The coronary artery tree was classified into 16 segments according to American Heart Association (AHA) classification [18]. The stenosis degree was visually classicized into four groups: normal, mild (<50% =, moderate (50% to 69%), severe (≥70%) [19, 20]. Coronary artery stenosis percentage was determined by comparison the luminal diameter of obstructive segment to the most normal appearing site proximal to the plaque [9]. If the image quality cannot provide accurate stenosis degree owing to motion artifacts, stairstep artifacts or the presence of calcification the segment was assigned to most appropriate group [19]. Moreover, the same 16-segment model was used for visually assessment of coronary atherosclerosis plaque which is tissue structures >1 mm^2^ existed either within the coronary artery lumen or adjacent to the coronary artery lumen that could be discriminated from surrounding tissue [19]. Plaques showing higher signal intensities than contrast enhanced lumen defined as calcified plaques (CP). Non-calcified plaques (NCP) were defined as any non-calcified stenosis >25% or any distinguishability structure in the coronary artery wall with a CT density less than contrast-enhanced coronary lumen but greater than surrounding tissues. Plaques meeting these requirements meanwhile showing calcification were classified as mixed plaques (MP) [21].

Based on above primary analysis, total plaque score (TPS), segment-stenosis score (SSS) and CAD severity (CADS) were used to illustrate the coronary atherosclerosis burden, and further clarify the spectrum of atherosclerosis (location, severity and characteristic of plaques) in coronary artery. 1) TPS was determined by summing the total number of segments exhibiting any stenosis or plaque irrespectively the degree of stenosis and the composition of the plaque which is ranging from 0 to 16, >5 points were defined as high-risk individual according to follow-up MACE (including ST-elevation myocardial infarction, non- ST-elevation myocardial infarction, acute coronary syndrome or cardiac death) [9, 10, 13]. 2) SSS was calculated as a measurement of total coronary plaque burden. Each segment was given a score from 0 to 3 [0 for normal, 1 for mild(<50%), 2 for moderate (50% to 69%), 3 for severe (≥70%) according to the degree of lumen stenosis. The summation of the every segment score received an entire score ranging from 0 to 48 >5 points were defined as high-risk individual according to follow-up MACE [9, 10, 13]. 3) According to coronary CTA imaging, subjects were divided into three coronary artery disease severity categories: normal (coronary arteries without any stenosis or plaque), non-obstructive CAD (coronary artery lumen stenosis <50% and any kinds of atherosclerosis plaque), or obstructive CAD (any coronary lumen lesions >50%) which is also defined as high-risk individual according to follow-up MACE [10, 14].

### Serum Lipid Test

On the same day of CTA performed, venous blood samples were obtained by venipuncture of large antecubital veins of participates after overnight fasting. The blood was centrifuged for blood test at hospital’s Department of Laboratory. Total cholesterol (TC), high density lipoprotein cholesterol (HDL-C), low density lipoprotein cholesterol (LDL-C), triglyceride (TG) and fasting blood-glucose (FBG) were determined using automatic biochemical analyzer and accessory kits (OLYMPUS AU5400, Olympus, Japan). TC and TG were measured by enzyme spectrophotometric colorimetry, LDL-C and HDL-C by double reagent direct method, FBG by enzymic method.

### Statistical Analysis

As TPS, SSS, CADS were skewed rather than normal distribution, median ± IQR (interquartile range) was used to describe their average and variation level, and nonparametric Kruskal-Wallis test (H-test) for multiple comparison test was further used to test difference between compared groups (gender, age groups, etc.). For intuitively visualizing the spectrum of atherosclerosis burden, both incidence and proportion of TPS and SSS were mapped onto the coronary artery tree based on American Heart Association (AHA) classification [18].

Furthermore, logistic regression model with gender & age adjusted was employed to identify independent predictors (lipid biomarkers, etc.) of atherosclerosis burden; the burden cut-off were defined as 1 for score >5 vs. 0 for score≤5 for TPS and SSS based on the follow-up of major adverse cardiac events (MACE) [9, 13], while 1 for CAD (non-obstructive or obstructive) vs. 0 for normal for CADS [14,15].

For all above involved hypothesis testing, a two-sided p value of <0.05 was considered statistically significant, and all analyses were performed with SAS 9.1.

Finely, receiver operating characteristic (ROC) curve was used to evaluate the discriminant effect for selected lipid predictors using the logistic regression model. The area under the curve (AUC) for the ROC analysis together with sensitivity, specificity, and criterion of probability of score >5 was calculated using MedCalc software for ROC curve analysis [22].

## Results

### Baseline Characteristics of Participants

Table 1 summarized the baseline characteristics of participants (S1 Data). It revealed the imbalance sample size between male and female dues to self-referred healthy check-up. Also the age rage covered youth, middle and old people for both male and female, most of them were greater than 45 years old (85.01%). The dominant occupation were civil service were (33.88%) and company employees were (54.72%) for both male and female groups. Furthermore, statistical significant differences between male and female were detected for hypertension, diabetes, obesity and smoke status respectively.

**Table 1:** Summarized on baseline characteristics of participants in this study.

Baseline characteristics	Categories	Female(n = 51)	Male(n = 256)	Total(n = 307)	P value
Age(years)	<45	9(17.65%)	37(14.45%)	46(14.98%)	<0.0001
	45–59	22(43.14%)	181(70.70%)	203(66.12%)	
	≥60	20(39.22%)	38(14.84%)	58(18.89%)	
Occupation	Civil Service	21(41.18%)	83(32.42%)	104(33.88%)	0.0216
	company employees	26(50.98%)	142(55.47%)	168(54.72%)	
	Bank staff	3(5.9%)	3(1.17%)	6(1.95%)	
	University Professors	1(1.96%)	28(10.94%)	29(9.45%)	
Hypertension		n = 25	n = 185	n = 210	
		7(28.00%)	68(36.76%)	75(35.71%)	0.5449
Diabetes		n = 26	n = 184	n = 210	
		3(11.56%)	17(9.24%)	20(9.52%)	0.7360
BMI(kg/m^2^)		n = 20	n = 155	n = 175	
	<24	7(35.00%)	41(26.45%)	48(27.43%)	0.5599
	24–27.9	10(50.00%)	76(49.03%)	86(49.14%)	
	≥28	3(15.00%)	38(24.52%)	41(23.43%)	
Smoker/ex-smoker		n = 51	n = 251	n = 302	
		1(1.96%)	123(49.00%)	124(41.05%)	<0.0001

### General Distribution of Coronary Artery Atherosclerosis Burden

**Fig 1:**
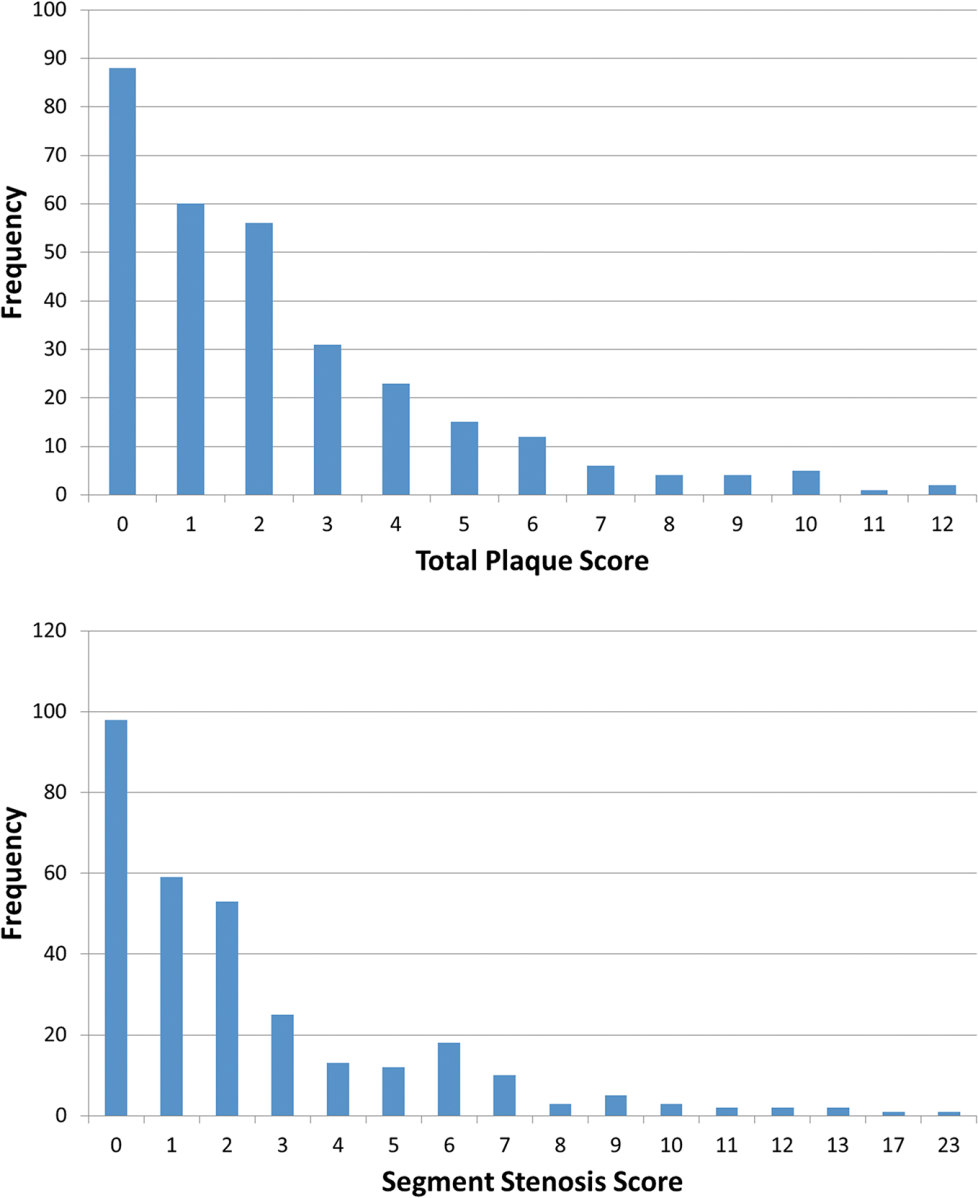
The frequency distribution of TPS and SSS score. TPS and SSS score were left skewness distribution. SSS = segment-stenosis score. TPS = total plaque score.

Fig 1 showed the general frequency distribution of TPS and SSS score, suggesting that both of them were extreme skewness distribution with their right long tail (S1 Data). For TPS (Table 2 and S1 Data), the median score was 2 with IQR of 3.5 for male and 1 with IQR of 2 for female (corresponding their mean score with deviation of 2.42±2.58 for male and 1.55±2.00 for female). The incidence of atherosclerosis plaque (score ≥1) was up to 71.34% (219/307) but just 11.07% (34/307) for score >5, indicating that the atherosclerosis plaque burden was serious with about one tenth as high risk individuals in this specific urban Han Chinese population. Similar trend (Table 2 and S1 Data) was also observed for SSS (median±IQR:2±3 for male and 1±2 for female, corresponding mean±deviation of 2.53±3.11 for male and 1.82±2.98 for female), with the incidence of 68.08% (209/307) for score ≥1, and of 15.31% (47/307) for score >5. For CADS (Table 2), incidence of stenosis was up to 71.34% (219/307), while obstructive CAD (any coronary lumen lesions >50%) reached 16.29% (50/307).

**Table 2:** The distribution of the scores in different gender and age groups.

			Total plaque score		Segment stenosis score		CAD severity n(%)		
		n	Mean±SD	Median±IQR	Mean±SD	Median±IQR	1	2	3
Gender	Female	51	1.55±2.00	1±2	1.82±2.98	1±2	21(41.18)	21(41.18)	9(17.65)
	Male	256	2.42±2.58	2±3.5	2.53±3.11	2±3	67(26.17)	148(57.86)	41(16.02)
	*χ* ^2^		-2.4385		-2.1618			-1.4312	
	P value		0.0147		0.0306			0.1524	
Age(year)	<45	46	1.61±1.86	1±3	1.61±2.18	1±2	19(41.3)	21(45.65)	6(13.04)
	45–59	203	2.26±2.64	1±3	2.33±3.15	1±3	61(30.05)	118(58.13)	24(11.82)
	≥60	58	2.86±2.37	2±3	3.33±3.36	2.5±4	8(13.79)	30(51.72)	20(34.48)
	*χ* ^2^		10.48		13.37			18.18	
	P value		0.0053		0.0013			0.0001	

Values are mean ± SD and median ± interquartile or n % (n/N). Nonparametric Kruskal-Wallis test (H-test) was used to test difference between genders and age groups.

CAD = coronary artery disease

### The Differences of Coronary Artery Atherosclerosis Burden in Gender and Age

As showed in Table 2, all TPS, SSS and CADS revealed significant differences between genders as well as age groups, with male much higher than female (*χ* ^2^ = -2.4385, P = 0.0147 for TPS; *χ* ^2^ = -2.1618, P = 0.0306 for SSS), and increasing trends with age (*χ* ^2^ = 10.48, P = 0.0053 for TPS; *χ* ^2^ = 13.37, P = 0.0013 for SSS), except no significant difference was observed between genders for CADS (*χ* ^2^ = -1.4312, P = 0.1524).

### Spectrum of Atherosclerosis Burden in Coronary Artery

**Fig 2:**
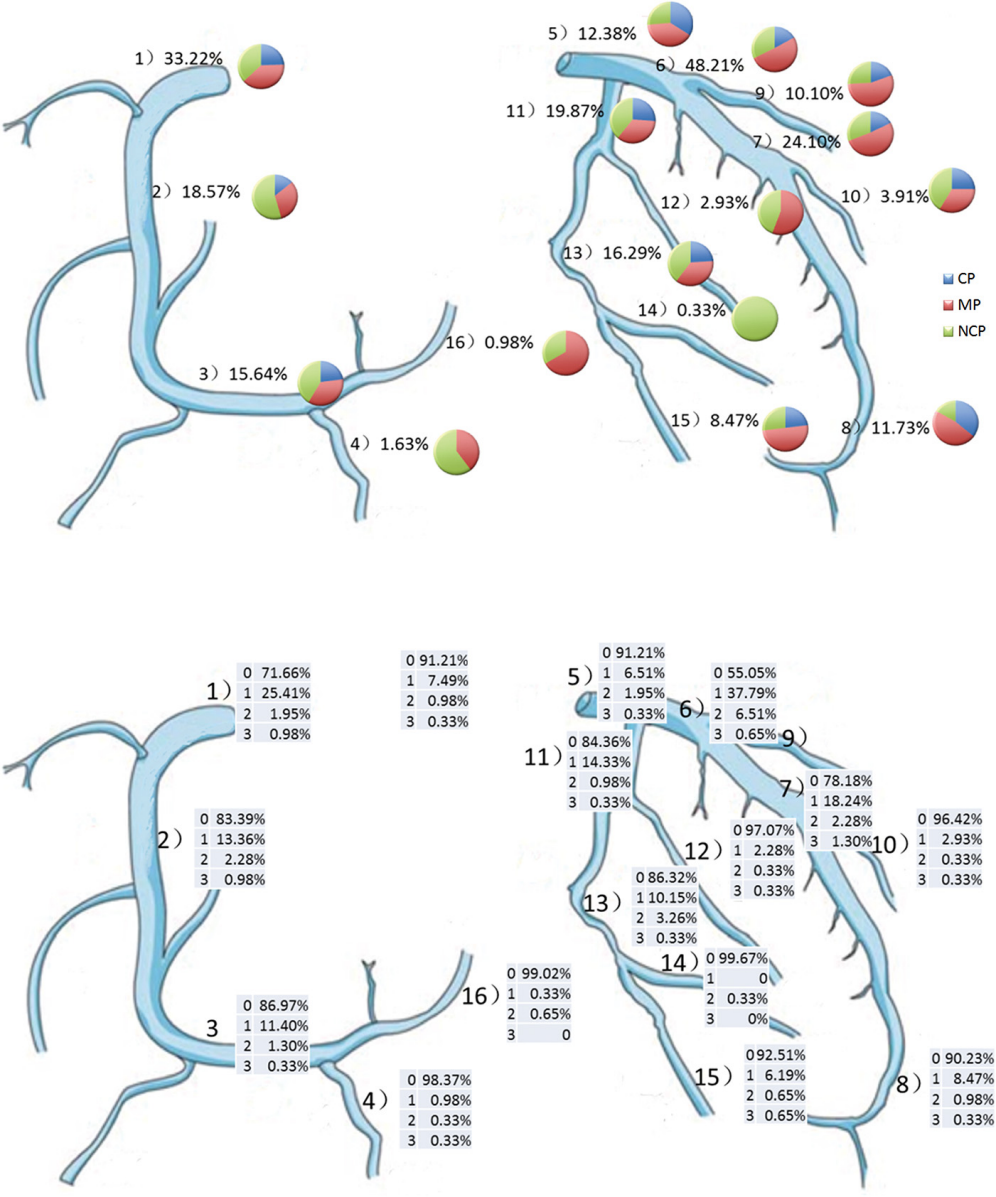
The spectrum of atherosclerosis burden in each segment of coronary artery tree. The coronary artery tree was classified into 16 segments according to American Heart Association classification 1] proximal, 2] mid, 3] distal right coronary artery; 4] posterior descending artery; 5] left main artery; 6] proximal, 7] mid, 8] distal left anterior descending artery; 9] first, 10] second diagononal branch; 11] proximal, 13] mid, 15] distal left circumflex artery; 12] first, 14] second obtuse marginal branch; 16] posterolateral ventricular branch]. The degree of stenosis and composition of plaque of each segment was evaluated. The incidence of plaque and proportion of plaque (CP, MP, NCP) were noted on each segment in fig 2 (top). The degree of stenosis of each segment was classicized into four groups: 0 for normal, 1 for <50%,2 for 50% to 69%, 3 for ≥70% and their proportion were recorded in fig 2 (lower). CP = calcified plaques. MP = mixed plaques. NCP = non-calcified plaques.

Fig 2 showed the spectrum of atherosclerosis burden (TPS on the top and SSS in the lower) in each segment by mapping their incidence & proportion onto the coronary artery tree (S1 Data). Obviously, for TPS, although plaque occurred on all 16 segments, the most lesion segment emerged on proximal left anterior descending artery (48.21%), followed by proximal right coronary artery (33.22%), mid left anterior descending artery (24.10%), proximal left circumflex artery (19.87%), and mid right coronary artery (18.57%), etc. Generally, among these high incidence lesion segments, the mixed plaque (MP) emerged as the most frequent type of plaque in left anterior descending artery, while non-calcified as dominant in right coronary artery and left circumflex artery. Similar pattern was also revealed for SSS (Fig 2 lower), with the most serious lesion segment on proximal left anterior descending artery (1: 37.79%, 2: 6.51%, 3: 0.65%), followed by proximal right coronary artery (1: 25.41%,2: 1.95%, 3: 0.98%), mid left anterior descending artery (1: 18.24%,2: 2.28%, 3: 1.3%), mid right coronary artery (1: 13.36%,2: 2.28%, 3: 0.98%), and proximal left circumflex artery (1: 14.33%,2: 0.98%, 3: 0.33%), etc.

### Association Analysis between Lipid Biomarkers and Atherosclerosis Burden Scores

Table 3 listed the results of logistic regression analysis for selecting lipid predictor to atherosclerosis burden with gender & age adjusted, suggesting that HDL-C was the significant predictor to both TPS [OR: 0.12 (0.02–0.82)] and SSS [OR: 0.15 (0.03–0.76)]. Unfortunately, no statistical significances were detected for TC, TG, LDL-C, FBG, BMI, hypertension, diabetes and obesity in this specific urban Han Chinese population.

**Table 3:** The results of logistic regression analysis of lipid predictor.

		Total plaque score	Segment stenosis score	CAD severity
	Mean±SD	OR(95%CI)	OR(95%CI)	OR(95%CI)
TG (mmol/L)	1.88±1.61	1.02(0.71 to 1.49)	1.07(0.79 to 1.47)	1.07(0.76 to 1.50)
TC (mmol/L)	5.12±0.96	0.62(0.38 to 1.01)	0.82(0.54 to 1.22)	1.18(0.78 to 1.79)
HDL—C (mmol/L)	1.37±0.32	0.12(0.02 to 0.82)	0.15(0.03 to 0.76)	0.55(0.14 to 2.21)
LDL—C (mmol/L)	3.11±0.75	0.70(0.38 to 1.29)	0.93(0.56 to 1.55)	1.36(0.80 to 2.31)
FBG (mmol/L)	5.74±1.32	1.14(0.87 to 1.48)	1.21(0.96 to 1.54)	1.02(0.78 to 1.35)
BMI (Kg/m^2^)	25.85±3.49	0.96(0.85 to 1.09)	0.97(0.87 to 1.09)	1.05(0.92 to 1.91)
Diabetes		0.55(0.12 to 2.66)	0.99(0.30 to 3.29)	1.36(0.43 to 4.34)
Hypertension		2.18(0.96 to 4.96)	1.67(0.80 to 3.50)	1.27(0.58 to 2.82)
Obesity		0.87(0.47 to 1.63)	0.81(0.45 to 1.43)	1.15(0.60 to 2.17)

Values are mean ± SD; OR = odds ratio; CI = confidence interval; CAD = coronary artery disease. TG = triglyceride; TC = total cholesterol; HDL-C = high density lipoprotein cholesterol; LDL-C = low density lipoprotein cholesterol; FBG = fasting blood-glucose; BMI = body mass index.

### Assessment of Discriminant Model for Serious Atherosclerosis Burden using HDL-C as Predictor

Based on above logistic regression analysis, two prediction models for the probability of TPS score >5 (or SSS score >5) could easily developed.

$$P_{TPS\;\text{score}>5}=\frac{\text{exp}(-3.457+1.2291sex+0.0586age-2.1146HDL)}{1+\exp(-3.457+1.2291sex+0.0586age-2.1146HDL)}$$

$$P_{\text{SSS score}>5}=\frac{\text{exp}(-2.6833+0.8511sex+0.0529age-1.8688HDL)}{1+\exp(-2.6833+0.8511sex+0.0529age-1.8688HDL)}.$$

**Fig 3:**
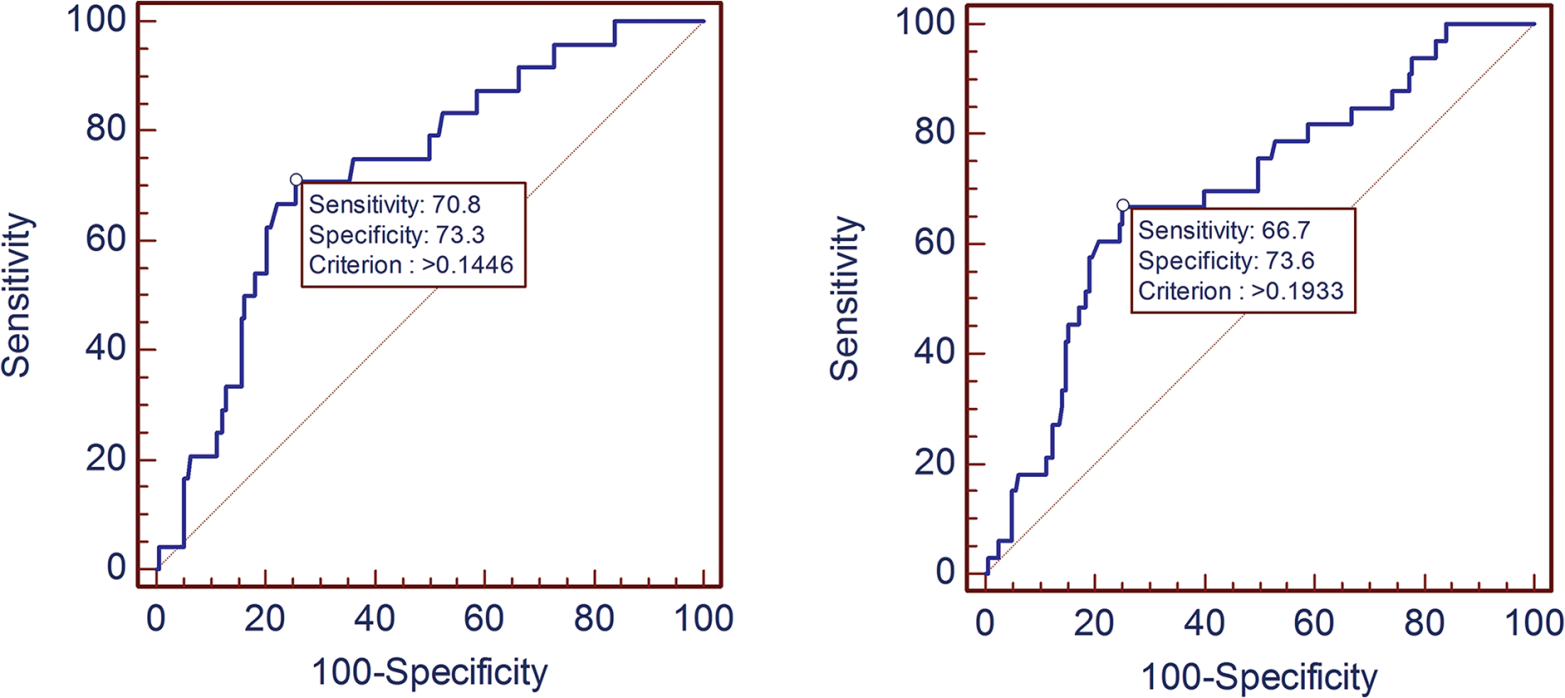
The ROC curve for predicting atherosclerosis burden. The ROC curve for predicting TPS score >5 in fig 3 (left), the AUC for TPS score >5 was 0.73 (95%CI 0.66–0.79). The ROC curve for predicting SSS score >5 in fig 3 (right), and the AUC for SSS score >5 was 0.70 (95%CI 0.63–0.76). AUC = area under the curve. ROC = receiver operating characteristic.

Fig 3 depicted the ROC curve for predicting atherosclerosis burden (for TPS score >5 in Fig 3 left, and for SSS score > 5 in Fig 3 right) with HDL-C as main predictor. The AUC for TPS score >5 was 0.73 (95%CI 0.66–0.79), and 0.70 (95%CI 0.63–0.76) for SSS score >5, suggesting that both models had good performance for identifying subjects with serious atherosclerosis burden.

## Discussion

In our result (Fig 1, Table 2 and S1 Data), the incidence of TPS score ≥1, SSS score ≥1,and CADS (non-obstructive and obstructive) were up to 71.34%, 68.08%, and 71.34%; and the proportion of high risk individuals [9, 13] reached up to 11.07%, 15.31% and 16.29% respectively. This indicated that the atherosclerosis plaque burden was serious with about one in ten as high risk individuals in this specific urban Han Chinese population. Similar high prevalence of atherosclerosis plaque had been detected in Taiwan general health check-up population (60%) [23], in Mediterranean area of Spain voluntary population (53%) [24]. However, in a South Koreans general routine health evaluation population [5], the prevalence of atherosclerosis plaque was only 21.5%. Therefore, different population might present different prevalence level of atherosclerosis plaque due to their different risk factors exposure background. The possible reasons of much higher burden of atherosclerosis plaque in this specific urban Han Chinese population were higher proportion of male (83.39%), older (53.29±8.69), working at office and self-referred, etc. Furthermore, all TPS, SSS and CADS revealed significant differences between genders as well as age groups, with male much higher than female, and increasing trends with age, except no significant difference was observed between genders for CADS (seeing Table 2). Similar differences of atherosclerosis plaque between genders were also reported in Taiwan [23], South Koreans [2] population, and United States and Europe [25], perhaps due to the protection of estrogen against coronary atherosclerosis [26]. The increased trend with age of atherosclerosis burdens observed in this study coincided to the fact that age is the obviously prognostic predictor for atherosclerosis plaque.

At present study, the whole spectrum of atherosclerosis burden was visualized on the coronary artery tree (TPS in Fig 2A and SSS in Fig 2B), suggesting that the most lesion segment emerged on proximal left anterior descending artery, followed by proximal right coronary artery, mid left anterior descending artery, proximal left circumflex artery, and mid right coronary artery, etc. Similar distributions of atherosclerosis lesion were also detected in suspected CAD patients, chest pain or dyspnea patients. For examples, in a known or suspected CAD of Europeans patients, the most lesion segment was located proximal artery and followed by medial portions of artery by magnetic resonance perfusion imaging [27]; a group of patient with chest pain or dyspnea in Dutch underwent coronary CTA showed the left anterior descending artery was the part of most lesions, followed by right and left circumflex artery [28]. The possible mechanism could be explained that hydrodynamic basis for early lesion development, which is locally disturbed flow, induces alterations that promote the steps of early atherosclerosis [29, 30]. Generally, the most emerged common lesion (Fig 2A) was mixed plaque (301, 42.94%), followed by non-calcified plaque (249, 35.52%) and calcified plaque (151, 21.54%). This revealed a quite difference from other self-referred asymptomatic healthy population in Taiwan with calcified plaque (44%) as dominant [23], and in South Koreans with non-calcified plaque (37.8%) [7] due to their different risk exposure. From the spectrum of coronary atherosclerosis (Fig 2A and Fig 2B), one can easily identify the incidence and characteristic of lesion in this specific urban Han asymptomatic healthy population. This was essential for early targeted intervention. Follow up study illustrated that subjects with proximal left anterior descending artery plaque exhibited higher of all-cause death [9], suggesting that early management of asymptomatic atherosclerosis is significant in clinical practice. Furthermore, the composition and size of plaque in asymptomatic healthy population is more significant than luminal narrowing, because the prediction of MACE is more important than just assessment the ischemia of myocardial, and the vulnerable plaque is usually associated with NCP [31]. Therefore, NCP could improve risk stratification and predict future cardiovascular events for asymptomatic individuals.

HDL-C was recognized as a significant predictor to both TPS [OR: 0.12 (0.02–0.82)] and SSS [OR: 0.15 (0.03–0.76)], and revealed a good performance for identifying subjects with serious atherosclerosis burden with AUC for TPS score >5 of 0.73 (95%CI 0.66–0.79), and 0.70 (95%CI 0.63–0.76) for SSS score >5 in this specific urban Han Chinese asymptomatic healthy population (Table 3 and Fig 3).Practically, HDL-C had been established a protective factor against coronary artery disease [32, 33, 34]. Theoretically, the major role of HDL-C in metabolism is reverse cholesterol transport which is cholesterol efflux from peripheral tissues to liver for biliary excretion [35, 36]. This metabolism process is the basis of prevention against CAD. Therefore, our findings were reasonable. In our study, no statistical significances were detected for TC, TG, LDL-C, FBG, BMI, hypertension, diabetes and obesity in this specific urban Han Chinese population. The possible reasons were the threshold of high-risk individuals and insufficient sample sizes. As there was no follow-up study of TPS and SSS in Chinese population currently, the threshold of high-risk individuals with TPS (score>5) and SSS (score>5) must only use the result of follow-up study in American population by James K. Min et al. this cutoff might not suitable for Chinese population [9]. In addition, only 212 subjects were used to detect the association between the serum lipid and atherosclerosis plaque burden dues to the financial insufficient in our study, large sample size cohort study of Chinese population will be conducted in our further work

### Limitations

Selection bias would be inevitable due to the subjects were self-referred and urban residents in routine health check-up. Further investigation should be conducted in large community population for highlighting the whole spectrum of coronary atherosclerosis burden of general asymptomatic healthy population. Only serum lipid was included as covariates for early predicting atherosclerosis burden, more biomarkers should be detected for improving performance of the model by follow up study.

## Conclusion

The atherosclerosis plaque burden was serious with about one in ten as high risk individuals in this specific urban Han Chinese population. On the whole spectrum of atherosclerosis burden, the most lesion segments emerged on proximal left anterior descending artery with mixed plaque as dominant. As potential surrogation of coronary CTA, HDL-C was recognized as a significant predictor to atherosclerosis burden and revealed a good performance for identifying high risk individuals.

## Supporting Information

S1 Data Coronary Computed Tomography Angiography and relevant information. (XLSX)

## References

[1] Rosamond W, Flegal K, Furie K, Go A, Greenlund K, Haase N, et al: Heart disease and stroke statistics—2008 update: a report from the American Heart Association Statistics Committee and Stroke Statistics Subcommittee. Circulation 117:e25, 2008 18086926 10.1161/CIRCULATIONAHA.107.187998

[2] Myerburg RJ, Interian A Jr., Mitrani RM, Kessler KM, Castellanos A. Frequency of sudden cardiac death and profiles of risk. J Am Coll Cardiol,1997;80:10F–9F. 10.1016/s0002-9149(97)00477-3 9291445

[3] Achenbach S. Computed tomography coronary angiography. J Am Coll Cardiol, 2006, 48(10): 1919–1928. 17112978 10.1016/j.jacc.2006.08.012

[4] Meijboom WB, Meijs MFL, Schuijf JD, Cramer MJ, Mollet NR, van Mieghem CA, et al Diagnostic Accuracy of 64-Slice Computed Tomography Coronary AngiographyA Prospective, Multicenter, Multivendor Study. J Am Coll Cardiol, 2008, 52(25): 2135–2144. 10.1016/j.jacc.2008.08.058 19095130

[5] Choi EK, Choi SI, Rivera JJ, Nasir K, Chang SA, Chun EJ, et al Coronary computed tomography angiography as a screening tool for the detection of occult coronary artery disease in asymptomatic individuals. J Am Coll Cardiol. 2008 7 29;52(5):357–65. 10.1016/j.jacc.2008.02.086 18652943

[6] Rivera JJ, Nasir K, Cox PR, Choi EK, Yoon Y, Cho I, et al Association of traditional cardiovascular risk factors with coronary plaque sub-types assessed by 64-slice computed tomography angiography in a large cohort of asymptomatic subjects. Atherosclerosis 2009 10; 206(2):451–7. 10.1016/j.atherosclerosis.2009.05.027 19524922

[7] Kim KJ, Choi SI, Lee MS, Kim JA, Chun EJ, Jeon CH. The prevalence and characteristics of coronary atherosclerosis in asymptomatic subjects classified as low risk based on traditional risk stratification algorithm: assessment with coronary CT angiography. Heart 2013; 99:1113–17. 10.1136/heartjnl-2013-303631 23723445

[8] Chang HJ, Chung N. Clinical perspective of coronary computed tomographic angiography in diagnosis of coronary artery disease. Circ J 2010; 75(2): 246–252. 10.1253/circj.cj-10-1206 21258164

[9] Min JK, Shaw LJ, Devereux RB, Okin PM, Weinsaft JW, Russo DJ, et al Prognostic value of multidetector coronary computed tomographic angiography for prediction of all-cause mortality. J Am Coll Cardiol 2007;50:1161–1170. 17868808 10.1016/j.jacc.2007.03.067

[10] Chow BJ, Wells GA, Chen L, Yam Y, Galiwango P, Abraham A, et al Prognostic value of 64-slice cardiac computed tomography severity of coronary artery disease, coronary atherosclerosis, and left ventricular ejection fraction. J Am Coll Cardiol 2010;55:1017–1028. 10.1016/j.jacc.2009.10.039 20202518

[11] Agatston AS, Janowitz WR, Hildner FJ, Zusmer NR, Viamonte MJr., Detrano R. Quantification of coronary artery calcium using ultrafast computed tomography. J Am Coll Cardiol 1990;15:827–32. 2407762 10.1016/0735-1097(90)90282-t

[12] O’ Rourke RA, Roundage B, Froelicher VF, Greenland P, Grundy SM, Hachamovitch R, et al: ACC/AHA Exper Consensus Document on electron-beam computed tomography for the diagnosis and prognosis of coronary artery disease. Circulation, 2000, 110(17): 2638–2643. 10.1161/01.cir.102.1.126 10880426

[13] Plank F, Friedrich G, Dichtl W, Klauser A, Jaschke W, Franz WM, et al The diagnostic and prognostic value of coronary CT angiography in asymptomatic high-risk patients: a cohort study. Open Heart, 2014, 1(1): e000096 10.1136/openhrt-2014-000096 25332810 PMC4189305

[14] Ostrom M P, Gopal A, Ahmadi N, Nasir K, Yang E, Kakadiaris I, et al Mortality incidence and the severity of coronary atherosclerosis assessed by computed tomography angiography. J Am Coll Cardiol, 2008, 52(16): 1335–1343. 10.1016/j.jacc.2008.07.027 18929245

[15] Hadamitzky M, Freißmuth B, Meyer T, Hein F, Kastrati A, Martinoff S, et al Prognostic value of coronary computed tomographic angiography for prediction of cardiac events in patients with suspected coronary artery disease. J Am Coll Cardiol Img, 2009, 2(4): 404–411. 10.1016/j.jcmg.2008.11.015 19580721

[16] Lawlor D A, Adamson J, Ebrahim S. Performance of the WHO Rose angina questionnaire in post-menopausal women: Are all of the questions necessary?[J]. Journal of epidemiology and community health, 2003, 57(7): 538–541. 12821705 10.1136/jech.57.7.538 PMC1732510

[17] Cooperative Meta-analysis Group of China Obesity Task Force. (2003) Predictive values of body mass index and waist circumference to risk factors of related diseases in Chinese adult population. Chin J Epidemiol 23: 5–10. 12015100

[18] Austen WG, Edwards JE, Frye RL, Gensini GG, Gott VL, Griffith LS, et al A reporting system on patients evaluated for coronary artery disease. Report of the Ad Hoc Committee for Grading of Coronary Artery Disease, Council on Cardiovascular Surgery, American Heart Association. Circulation 1975; 51(4):5–40. 1116248 10.1161/01.cir.51.4.5

[19] Hoffmann U, Moselewski F, Cury RC, Ferencik M, Jang IK, Diaz LJ, et al Predictive value of 16-slice multidetector spiral computed tomography to detect significant obstructive coronary artery disease in patients at high risk for coronary artery disease patient-versus segment-based analysis. Circulation, 2004, 110(17): 2638–2643. 15492297 10.1161/01.CIR.0000145614.07427.9F

[20] Achenbach S. Quantification of Coronary Artery Stenoses by Computed Tomography. J Am Coll Cardiol Img, 2008, 1(4): 472–474. 10.1016/j.jcmg.2008.05.008 19356469

[21] Hadamitzky M, Täubert S, Deseive S, Byrne RA, Martinoff S, Schömig A, et al. Prognostic value of coronary computed tomography angiography during 5 years of follow-up in patients with suspected coronary artery disease. Eur Heart Journal, 2013: eht293. 10.1093/eurheartj/eht293 24067508

[22] Normann J, Mueller M, Biener M, Vafaie M, Katus HA, Giannitsis E. Effect of older age on diagnostic and prognostic performance of high-sensitivity troponin T in patients presenting to an emergency department. Am Heart J 164: 698–705 e694. 10.1016/j.ahj.2012.08.003 23137500

[23] Lee BC, Lee WJ, Hsu HC, Chien KL, Shih TT, Chen MF. Using clinical cardiovascular risk scores to predict coronary artery plaque severity and stenosis detected by CT coronary angiography in asymptomatic Chinese subjects. Int J Cardiovas Imag, 2011, 27(5): 669–678. 10.1007/s10554-011-9874-6 21695485

[24] Descalzo M, Leta R, Rosselló X, Alomar X, Carreras F, Pons-Lladó G. Subclinical Coronary Atherosclerosis Identified by Coronary Computed Tomography Angiography in Asymptomatic Population by Coronary Artery Disease Risk Level. Revista Española de Cardiología, 2013, 66(06): 504–505. 24776058 10.1016/j.rec.2012.12.012

[25] Lansky AJ, Ng VG, Maehara A, Weisz G, Lerman A, Mintz GS, et al Gender and the extent of coronary atherosclerosis, plaque composition, and clinical outcomes in acute coronary syndromes. J Am Coll Cardiol Img, 2012, 5(3s1): S62–S72. 10.1016/j.jcmg.2012.02.003 22421232

[26] Burke AP, Farb A, Malcom G, Virmani R. Effect of menopause on plaque morphologic characteristics in coronary atherosclerosis. Am Heart J 2001; 141:S58–62. 11174360 10.1067/mhj.2001.109946

[27] Manka R, Paetsch I, Kozerke S, Moccetti M, Hoffmann R, Schroeder J, et al Whole-heart dynamic three-dimensional magnetic resonance perfusion imaging for the detection of coronary artery disease defined by fractional flow reserve: determination of volumetric myocardial ischaemic burden and coronary lesion location. Eur Heart Journal, 2012, 33(16): 2016–2024. 10.1093/eurheartj/ehs170 22677136

[28] De Graaf MA, Broersen A, Ahmed W, Kitslaar PH, Dijkstra J, Kroft LJ, et al Feasibility of an Automated Quantitative Computed Tomography Angiography–Derived Risk Score for Risk Stratification of Patients With Suspected Coronary Artery Disease. Am J Cardiol, 2014, 113(12): 1947–1955. 10.1016/j.amjcard.2014.03.034 24798123

[29] Gimbrone MA, Resnick N, Nagel T, Khachigian LM, Collins T, Topper JN. Hemodynamics, Endothelial Gene Expression, and Atherogenesisa. Ann NY ACAD Sci, 1997, 811(1): 1–11. 10.1111/j.1749-6632.1997.tb51983.x 9186579

[30] Gijsen F, van der Giessen A, van der Steen A, Wentzel J. Shear stress and advanced atherosclerosis in human coronary arteries. J Biomech, 2013, 46(2): 240–247. 10.1016/j.jbiomech.2012.11.006 23261245

[31] Maurovich-Horvat P, Ferencik M, Voros S, Merkely B, Hoffmann U. Comprehensive plaque assessment by coronary CT angiography. Nat Rev Cardiol, 2014. 10.1038/nrcardio.2014.60 24755916

[32] Gordon T, Castelli WP, Hjortland MC, Kannel WB, Dawber TR. High density lipoprotein as a protective factor against coronary heart disease: the Framingham Study. Am J Med, 1977, 62(5): 707–714. 193398 10.1016/0002-9343(77)90874-9

[33] Di Angelantonio E, Sarwar N, Perry P, Kaptoge S, Ray KK, Thompson A, et al Major lipids, apolipoproteins, and risk of vascular disease. JAMA, 2009, 302(18): 1993–2000. 10.1001/jama.2009.1619 PMC3284229 19903920

[34] Huxley RR, Barzi F, Lam TH, Czernichow S, Fang X, Welborn T, et al Isolated Low Levels of High-Density Lipoprotein Cholesterol Are Associated With an Increased Risk of Coronary Heart Disease An Individual Participant Data Meta-Analysis of 23 Studies in the Asia-Pacific Region. Circulation, 2011, 124(19): 2056–2064. 10.1161/CIRCULATIONAHA.111.028373 21986289

[35] Lewis GF, Rader DJ. New insights into the regulation of HDL metabolism and reverse cholesterol transport. Circ Res, 2005, 96(12): 1221–1232. 15976321 10.1161/01.RES.0000170946.56981.5c

[36] Hellerstein M, Turner S. Reverse cholesterol transport fluxes. Curr Opin Lipdol, 2014, 25(1): 40–47. 10.1097/MOL.0000000000000050 24362356

